# An inventory of weed diversity data including invasive alien species as emerging threats to agro-ecosystem

**DOI:** 10.1016/j.dib.2026.113179

**Published:** 2026-08-18

**Authors:** Rajeshwary S R, Varshitha G M, Abhishek Pujari, Vinutha S Asundi, Chethan Prasad M, Rashmi Shivanna

**Affiliations:** a Post Graduate Department of Botany, JSS College of Arts, Commerce and Science (Autonomous), Ooty road, Mysuru, Karnataka, India; b Manipal Institute of Technology Bengaluru, Manipal Academy of Higher Education, Manipal, India

**Keywords:** Agricultural challenge, Mysore district, Crop fields, Invasive alien species, Ethnobotany, Medicinal uses, *Parthenium hysterophorus* L*.*

## Abstract

Weed invasion has emerged as one of the foremost ecological and agricultural challenges, undermining crop productivity and threatening native biodiversity. Agricultural landscapes, particularly in regions of high cultivation intensity, provide ideal conditions for establishing and spreading invasive alien plants. Weed flora was surveyed across ten cultivated fields at six sites in Mysore district, Karnataka, India with monthly visits over 12 months; 1-hectare focal fields; 4 quadrats per field to document species diversity and assess invasive potential. About 51 species was recorded (42 genera, 22 families); Asteraceae (n = 11) and Poaceae (n = 10) were dominant. *Parthenium hysterophorus* L. was the most widespread (present in 9/10 fields). Using authoritative databases, we classified 15 species as invasive (29.41%). Ethnobotanical interviews indicated mixed local perceptions: several species have medicinal uses that complicate management. Ecological traits that facilitate invasion and recommend targeted early detection, community-based monitoring, and integrated weed management tailored to smallholder cropping systems.

Specifications Table

**Table:** 

Subject	Earth & Environmental Sciences
Specific subject area	*Weed ecology and invasive alien species in smallholder cropping systems*
Type of data	Table and Figure. Raw, Analyzed, Filtered, Processed
Data collection	Weed flora was surveyed across ten cultivated fields at six sites in Mysore district, with monthly visits over 12 months; 1-hectare focal fields; 4 quadrats per field to document species diversity and assess invasive potential.
Data source location	Krishnarajanagar and surrounding agricultural landscapes within Mysore district, Karnataka, India (sites: 12.445675°N, 76.390040°E; 12.448291°N, 76.390405°E; 12.441995°N, 76.386488°E; 12.450455°N, 76.384732°E; 12.441961°N, 76.393846°E; 12.453467°N, 76.388146°E).
Data accessibility	Repository name: Zenodo Data identification number: DATASET OF WEED DIVERSITY AND INVASIVE ALIEN SPECIES Direct URL to data: https://doi.org/10.5281/zenodo.21253808 Instructions for accessing these data: Open to public
Related research article	None

## 1 Value of the Data

• The data serves as a baseline inventory for weed flora in Mysore, essential for monitoring changes in biodiversity and the spread of invasive species over time.
• It gives important information for Integrated Weed Management (IWM), which is specifically designed for smallholder farming systems in tropical areas.
• The documented ethnobotanical uses give us an idea of how local communities see weeds, which is important for coming up with management plans that are both effective and socially acceptable.
• The data assists in the early detection of invasive species, such as *Parthenium hysterophorus* L, which poses threats to both crop production and native biodiversity

## 2 Background

The dataset was created to keep track of the different types of weeds and the presence of invasive alien plant species in smallholder farms in Mysore district, Karnataka, India. The impetus stems from the growing acknowledgment of biological invasions as a significant ecological phenomenon affecting agro-ecosystems, especially in the context of intensified agriculture and landscape alteration. The research is based on invasion ecology frameworks that view invasions as staged processes and stress the significance of local-scale inventories for prompt detection and monitoring.

Methodologically, the dataset was produced via systematic floristic surveys performed across ten cultivated fields, each representing various crop types. The sampling method was based on quadrats within 1-hectare focal fields, with monthly observations over a 12-months period (November 2021–October 2022) to see how species presence changed over time. We used regional floras and herbarium comparisons to figure out what species they were, and we used global databases like Plants of the World Online (POWO, Kew Science) [1] and the Global Biodiversity Information Facility (GBIF) [2] to make sure the names were correct. We recorded other characteristics, such as habit, nativity, crop association, and ethnobotanical uses, through field observations and semi-structured interviews with local farmers.

## 3 Data Description

In the present study, the diversity of weed species and their potential invasiveness were documented from selected crop landscapes of Mysore district, Karnataka, India. Weed species were collected from 10 different crop fields across 40 different quadrat samples of six field sites. A total of 51 species, representing 42 genera of 22 families, were recorded as weeds during the survey from 10 different crop fields, out of which 15 species are found to be an invasive species, that is 29.41% of total weed diversity documented during the survey.

**Fig. 1:**
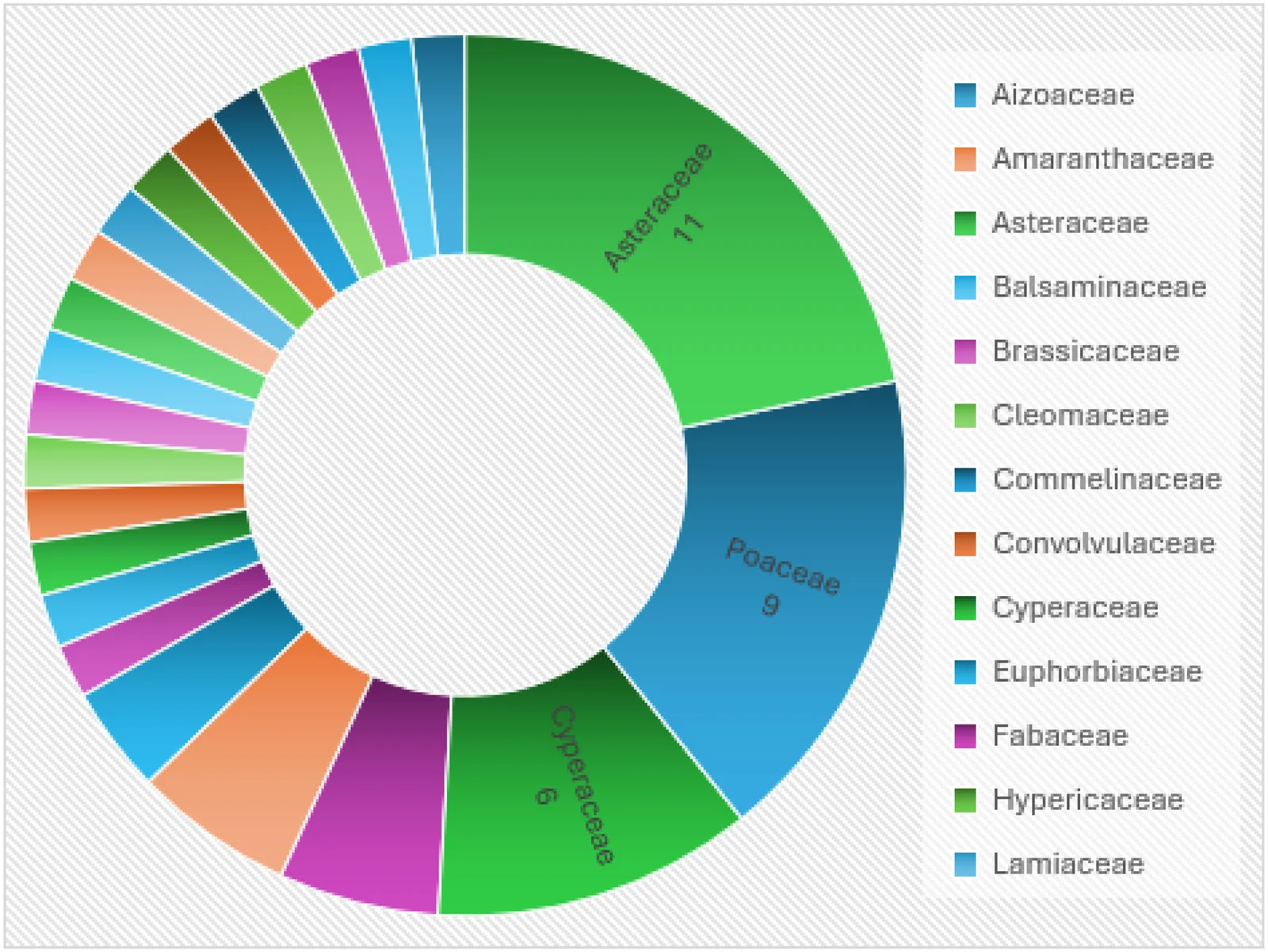
Family composition of weed species.

**Fig. 2:**
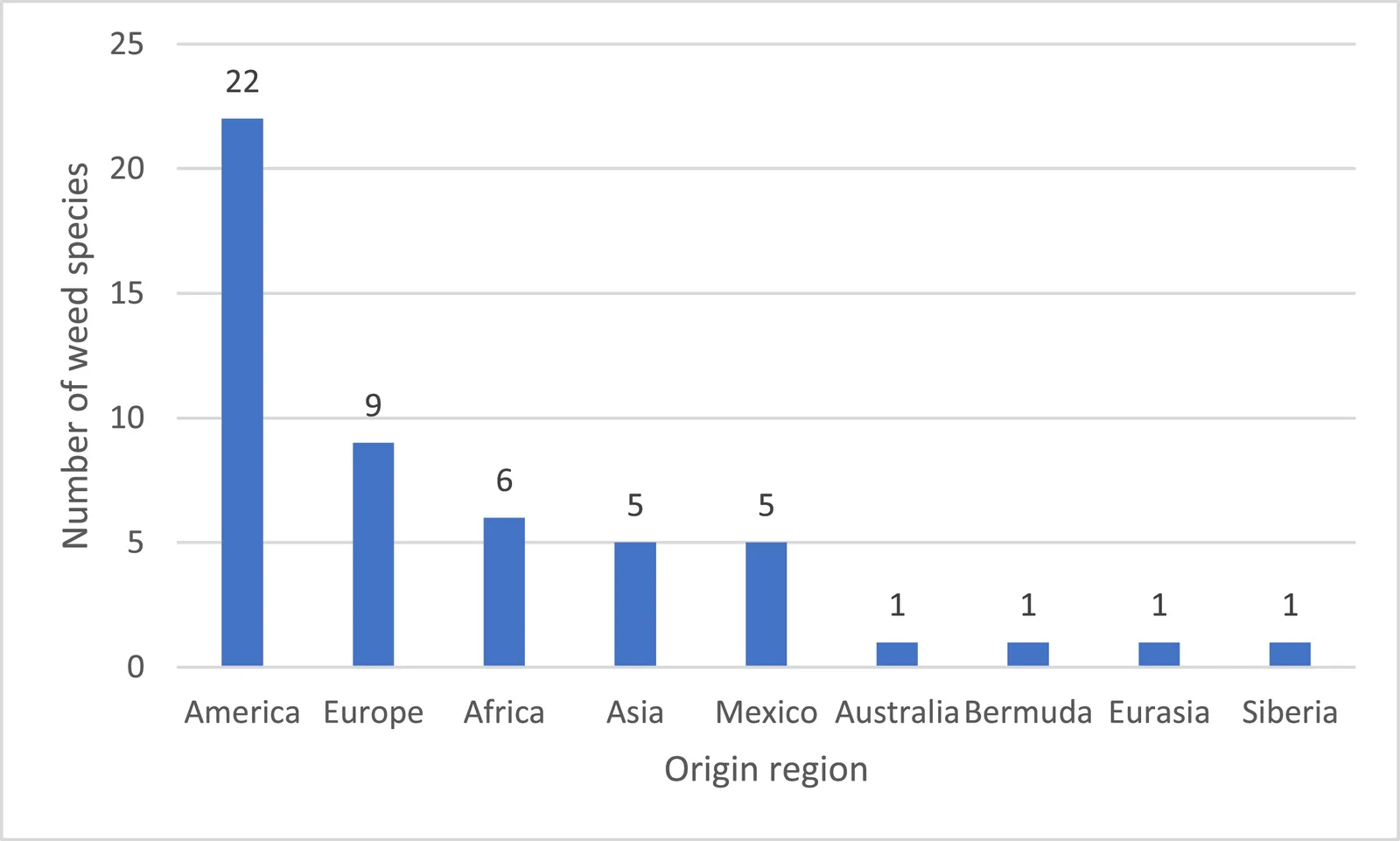
Origin regions of weed species.

**Fig. 3:**
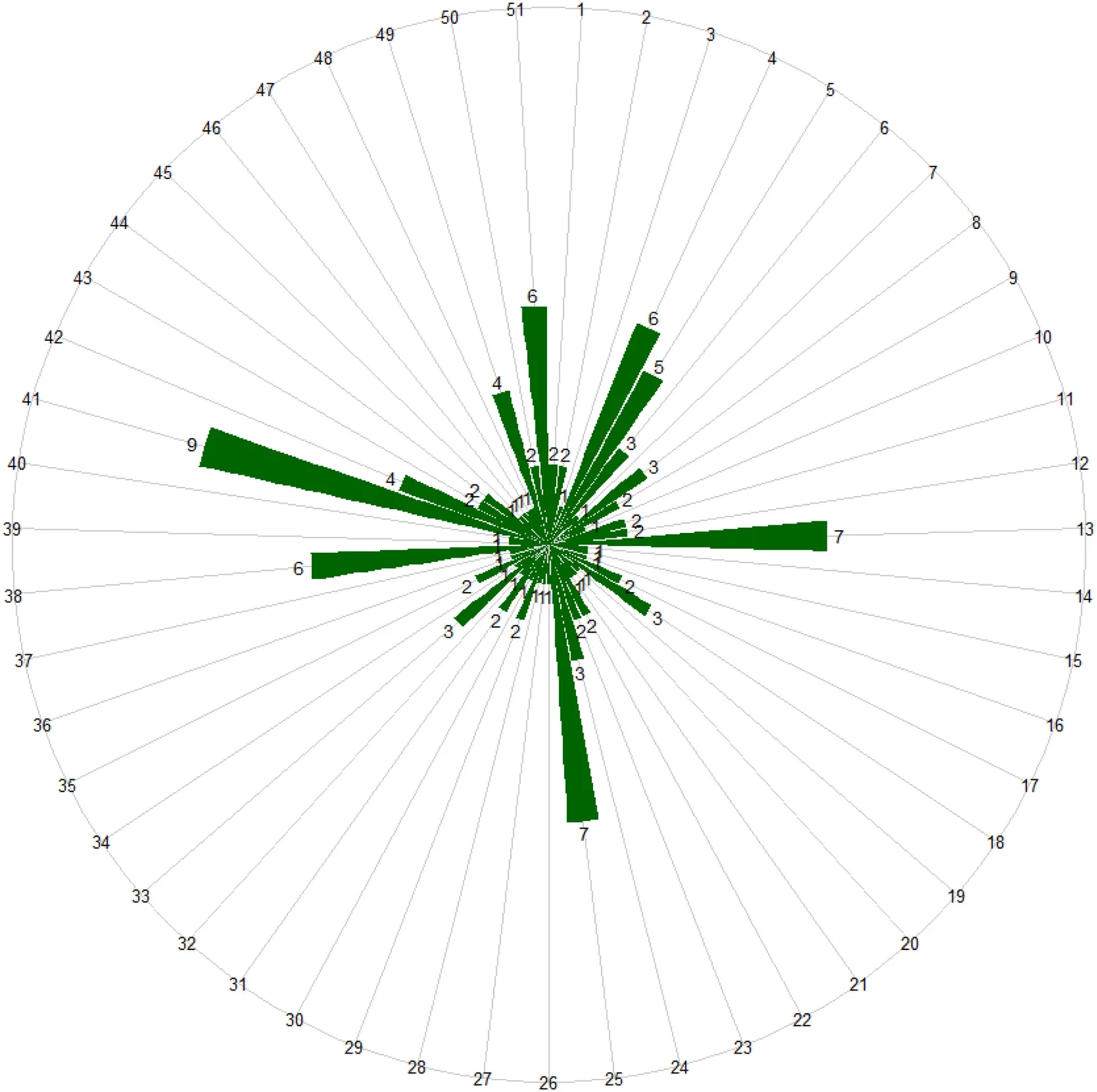
Circle bar plot of the occurrence of weeds across crop fields. Numbers on the outer circle corresponds to the species listed in Table 2, while inner circle represent number of different crop fields in which each weed species occurred.

Analysis of weed diversity across crop fields revealed that finger millet (*Eleusine coracana* (L.) Gaertn) supported the highest weed diversity, followed by chilli (*Capsicum annuum* L.), paddy (*Oryza sativa* L.), and tomato (*Solanum lycopersicum* L.) (Table 1). At the family level, Asteraceae accounted for the maximum number of weed species (11), followed by Poaceae (10), Cyperaceae (6), Fabaceae, and Amaranthaceae (3 species each). The remaining families were represented by a single species each (Fig. 1). Regarding biogeographical origin, most invasive weeds were introduced from America, Europe, and Mexico (Fig. 2). Among the recorded taxa, *Parthenium hysterophorus* L. was the most widespread species, occurring in nine out of the ten crop fields studied, followed by *Euphorbia hirta* L. and *Cynodon dactylon* (L.) Pers., which were also frequently encountered (Table 2 and Fig. 3).

**Table 1:** Weed diversity across major crop fields.

CROP	NUMBER OF WEED SPECIES
Finger millet	17
Chilli	16
Paddy	15
Tomato	15
Banana	13
Okra	13
Brinjal	8
Sugar cane	8
Bitter guard	7
Corn	7

**Table 2:** Weeds species in different crop fields.

SL NO of species	Name of the weed species	SL NO of species	Name of the weed species
1	*Abutilon theophrasti* Medik.	27	*Fimbristylis miliacea* L.
2	*Acmella paniculata* Delile	28	*Galinsoga quadriradiata* L.
3	*Agrostis capillaris* L.	29	*Gompherena serrata* L.
4	*Amaranthus retroflexus* L.	30	*Helianthus tuberosus* L.
5	*Amaranthus spinosus* L.	31	*Hypericum perforatum* L.
6	*Artemisia dracunculus* L*.*	32	*Hyptis suaveolens* L.
7	*Bidens pilosa* L*.*	33	*Impatiens capensis* meerb
8	*Chloris barbata* L.	34	*Imperata cylindrica* L.
9	*Chromolaena odorata* L.	35	*Ludwigia octovalvis (jacq)* Raven
10	*Cleome viscosa* L.	36	*Malvastrum coromandelianum* (L.) Garcke
11	*Commelina benghalensis* L.	37	*Marsilea qudrifolia* L.
12	*Cyanthillium cinereum* L.	38	*Mimosa pudica* L.
13	*Cynodon dactylon* L.	39	*Oldenlandia corymbosa* L.
14	*Cyperus brevifolia* Hassk.	40	*Panicum virgatum* L.
15	*Cyperus diffornis* L.	41	*Parthenium hysterophorus* L.
16	*Cyperus esculantus* L.	42	*Physalis angulata* L.
17	*Cyperus microiria* Steud	43	*Portulaca oleracea* L.
18	*Dactyloctenium aegyptum* L.	44	*Scoparia dulcis* L.
19	*Descurainia sophia* L.	45	*Senna obtusifolia* L.
20	*Dichondra carolinensis* michx.	46	*Sesbania cannabina* (Retz.) Poir.
21	*Digitaria sanguinalis* L.	47	*Setaria pumila* (Poir.) Roem. & Schult.
22	*Eclipta prostate* L.	48	*Setaria verticillata* L.
23	*Erigeron bonariensiss* L.	49	*Stachytarpheta jamaicensis* L.
24	*Euphorbia heterophyll* L.	50	*Synedrella nodiflora* L.
25	*Euphorbia hirta* L.	51	*Trianthema portulastrum* Linn.
26	*Fimbristylis dichotoma* L**.**	——–	——————————————–

## 4 Experimental Design, Materials and Methods

### 4.1 Field survey

Field surveys were carried out in Krishnarajanagar and the surrounding agricultural landscapes of Mysore district, Karnataka, India (12.445675°N, 76.390040°E; 12.448291°N, 76.390405°E; 12.441995°N, 76.386488°E; 12.450455°N, 76.384732°E; 12.441961°N, 76.393846°E; and 12.453467°N, 76.388146°E). The region is characterized by predominantly red loamy soils, an average annual rainfall of approximately 799 mm, and a mixed smallholder farming system. Ten agricultural fields representing different crop types were selected for the study, with each field covering approximately 1 ha. Vegetation surveys were conducted monthly over a 12-month period from November 2021 to October 2022.

Within each field, four randomly selected quadrats of “10×10 m” were established, with a spacing of between adjacent quadrats so they don’t overlap. The same quadrats were revisited throughout the study during each monthly survey. All non-crop vascular plant species occurring within the quadrats were recorded. The vegetation data obtained from the four quadrats were for subsequent analyses.

### 4.2 Collection and identification of plant material

Floristic surveys were conducted in fields cultivated with chilli (*Capsicum annuum* L.), paddy (*Oryza sativa* L.), finger millet (*Eleusine coracana* (L.) Gaertn.), okra (*Abelmoschus esculentus* (L.) Moench*.*), yard-long bean (*Vigna unguiculata subsp. Sesquipedalis* (L.) Verdc*.*), maize (*Zea mays* L.), banana (*Musa* spp.), sugarcane (*Saccharum officinarum* L.), tomato (*Solanum lycopersicum* L.), and brinjal (*Solanum melongena* L.). All non-crop vascular plant species encountered within the fields and along their margins were collected for identification.

Collected specimens were transported to the botany laboratory and processed following standard herbarium techniques, including pressing, drying, mounting, and labelling. Voucher specimens were deposited in the JSS Herbarium (Herbarium code: JSS). Species identification was carried out using regional floras [[3], [4], [5]] and verified through comparison with authenticated herbarium specimens. Current accepted names, synonyms, and author citations were checked using [[1], [2]].

To supplement the field observations, semi-structured interviews were conducted with 38 farmers and knowledgeable residents from the study area. The interviews focused on documenting vernacular names, local uses, and farmers perceptions of weed species. Prior informed consent was obtained from all participants, and the information collected was used to verify the local status of weed species alongside published floras and relevant literature. Interview responses were recorded anonymously and used only for validation purposes.

The nativity, geographical origin, and invasion status of each recorded species were determined using authoritative taxonomic references and published literature on the invasive alien flora of India. Invasive species were further classified following the framework proposed by [[6], [7]]. Information on taxonomy, nativity, origin, and invasion status was systematically compiled for all recorded species.

## Limitations

The studies have concentrated on the diversity and species dynamics of weeds; further investigation is required into the weed survival strategies, including dispersal parameters and associated soil physiography.

## CRediT Author Statement

**Rajeshwary S R:** Conceptualization, Methodology and Format analysis. **Varshitha G M:** Data curation, writing, original draft preparation. **Abhishek Pujari:** Visualisation, Writing, Reviewing and Editing. **Vinutha S Asundi:** Investigation, Writing, Reviewing and Editing. **Chethan Prasad M:** Format analysis, Writing, Reviewing and Editing. **Rashmi S:** Supervision, Conceptualization, Reviewing and Editing

## Ethics Statement

The authors have read and follow the ethical requirements for publication in Data in Brief and confirm that the current work does not involve human subjects, animal experiments, or any data collected from social media platforms.

## Data Availability

ZenodoJuly 8, 2026 (v2)DatasetOpen DATASET OF WEED DIVERSITY AND INVASIVE ALIEN SPECIES (Original data).
